# Patient-Led Peer Support Groups Improve Anti-Hypertensive Drugs Access For Adults Living With HIV And Hypertension In Rural Uganda – A Cross-Sectional Study

**DOI:** 10.21203/rs.3.rs-6445280/v1

**Published:** 2025-06-18

**Authors:** Mucunguzi Atukunda, Brian Twinamatsiko, Michael Ayebare, Elizabeth Arinitwe, Aida N Kawuma, Ronald Kiguba, Joan Nangendo, Gerald Mutungi, Fred C. Semitala, Moses R. Kamya, Jane Kabami

**Affiliations:** 1 Infectious Diseases Research Collaboration (IDRC) Uganda; 2 Uganda Heart Institute (UHI) Uganda; 3 Infectious Diseases Institute, Makerere University; 4 Makerere University, Kampala, Uganda; 5 Ministry of Health (MoH) Uganda

**Keywords:** HIV, Hypertension, Peer Support groups, Anti-Hypertensive drugs, multi-morbidity

## Abstract

**Introduction:**

People living with HIV (PLHIV) often have poorly controlled hypertension due to medication non-adherence, primarily caused by limited access to antihypertensive drugs. Peer support groups are being explored to improve access. We evaluated the feasibility and acceptability of peer support groups to improve access to anti-hypertensive drugs for PLHIV with hypertension.

**Methods:**

From December 2022 to April 2023, we conducted a cross-sectional survey and measurement of blood pressure among PLHIV in 26 health facilities to assess the accessibility of anti-hypertensive drugs in the peer support groups. The collected data was summarized using descriptive statistics

**Results:**

Eight out of 26 health facilities formed peer support groups. Among 163 PLHIV interviewed only 64 participated in the peer support groups. Among those who participated in peer support groups 57% reported accessing anti-hypertensive drugs through the group. The peer support groups were affordable for most participants. Around 60% found the contributions manageable, with 79% contributing $1.3 or less. Most participants expressed confidence in the performance of the peer support groups.

**Conclusion:**

The results of the study suggest that patient-led peer support groups are a feasible intervention to improve access to hypertensive medications for PLHIV with Hypertension.

## Introduction

Hypertension among people living with HIV (PLHIV) shows a growing trend globally (1). With the introduction of Anti-retroviral therapy (ART), the life expectancy of PLHIV has substantially improved (2). As a result of ageing, PLHIV are at a higher risk of suffering multi-morbidity including HTN (3). The prevalence of hypertension among PLHIV ranges from 17–50% in low- and middle-income countries(4-8). The management of PLHIV with hypertension includes lifestyle modifications and the use of anti-hypertensive medicines(9). The use of anti-hypertensive drugs is the mainstay treatment of uncontrolled Hypertension. However, Hypertension control among PLHIV is sub-optimal (10, 11) and non-adherence to anti-hypertensive medicine remains the leading cause of sub-optimal Hypertension control among Hypertension patients (12). One of the key drivers of non-adherence is limited access to drugs because of high costs, lack of knowledge among health providers, and clinical inertia. The World Health Organization (WHO) calls for increased access and affordability of all medicines (13). However, access to drugs to prevent and treat non-communicable diseases (NCDs) is unacceptably low in Uganda (14-18). The inadequacy of stocks of medicines for NCDs affects all hypertension patients including PLHIV(19, 20). Thus, low- and middle-income countries are encouraged to increase mobilization of domestic resources to cater for the many patients with NCDs with limited access to anti-hypertensives (21). Even then, national government mobilization is limited and the burden of access to anti-hypertensive medicines is largely left to patients to make out-of-pocket payments.

Peer support groups have been instrumental in providing support to patients with chronic illnesses (22). Peer-to-peer engagements create opportunities for support in areas such as adherence, counselling, patient education and patient advocacy (23). In the case of Uganda, the Ministry of Health (MoH) supports the formation of patient associations to tackle the issue of drug stockouts. In that regard, MoH issued guidance to healthcare managers allowing the formation of peer support groups. The peer support groups ask members to make voluntary financial contributions to purchase anti-hypertensive drugs in bulk at a low cost. This ensures that all patients in the support group access anti-hypertensive medicines during routine clinic visits to the health facility.

HIV-related clinical activities offered at the health facilities included HIV testing counselling and management of ART. Provision of HIV services at these health facilities is made by, medical doctors, clinical officers, nurses, HIV counsellors, laboratory technicians, pharmacy technicians, and record officers, supported by HIV expert clients. HTN care and treatment services are routinely offered at all these health facilities. If a PLHIV has Hypertension, the clinician prescribes both ART and Hypertension medicines and gives the individual a future appointment for clinical evaluation and monitoring. All available drugs and services at the health facility are provided at no cost to the patients. However, during drug stock-out periods, patients are advised to purchase medicines at private pharmacies of their preference. The work detailed in this manuscript was performed alongside that of the Integrated HIV/HTN project a cluster randomized trial (https://classic.clinicaltrials.gov/ct2/show/NCT04624061). As part of the Integrated HIV/HTN project implementation, project staff recommended establishing peer support groups at health facilities, aligning with Ministry of Health guidance. The Integrated HIV/HTN project aimed to measure the effectiveness of integration on HTN screening, blood pressure control and dual HIV and HTN control among PLHIV. We evaluated the feasibility and acceptability of patient-led peer support groups to improve anti-hypertensive drug stockouts for PLHIV with HTN within the Integrated HIV/HTN project.

## Methods

### Study design

We conducted a cross-sectional survey using a structured questionnaire to a randomly selected PLHIV with HTN attending peer support groups and PLHIV with HTN not attending peer support groups from 1 December 2022 to 28 April 2023.

### Study setting and population

**Figure 1:**
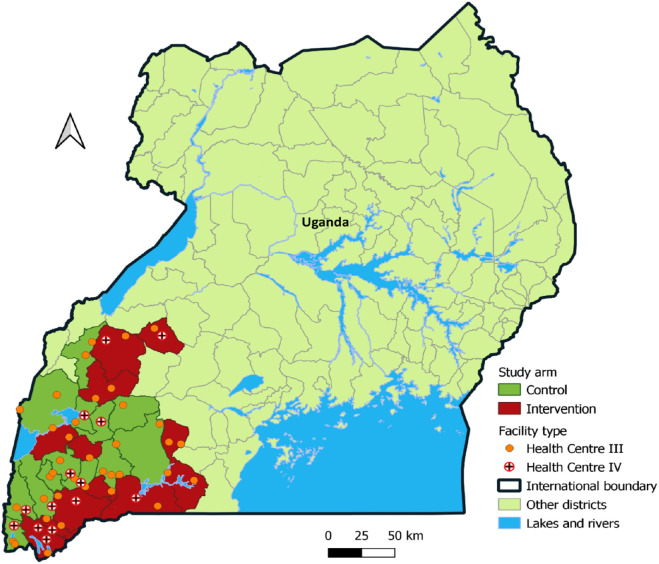
Map of Uganda showing the locations of study sites within selected districts in the southwestern region that participated in the cross-sectional survey December 2022 to April 2023. (Grey boundaries represent Districts)

The study was conducted at intervention health facilities under the Integrated HIV/HTN project in Southwestern Uganda (See Fig. 1). There were 26 government-supported health facilities under the intervention providing comprehensive HIV services with the number of PLHIV at the health facility ranging from 200–1,000 persons. The intervention package components included (1) Training and capacity building on the Integrated HIV/HTN model and NCD care. (2) The Integrated HIV/HTN care delivery model by promoting HTN screening and care in HIV clinics. (3) HMIS enhancements through mentorship and coaching on the use of NCD registers and patient cards, and capture of HTN data in the existing Electronic Medical Records (EMR) of Uganda; and (4) SMS and/or WhatsApp messages for data coordination and communication among providers, District Health Officers, and project staff. The integrated HIV/HTN protocol has been published (24).

### Formation of peer support groups:

The concept of peer support groups was introduced to the study facilities by the Integrated HIV/HTN project team during a performance review meeting attended by superintendents of all the participating health facilities in April 2021. This followed reports from healthcare providers indicating that many health facilities experienced frequent stockouts of anti-hypertensive medicines due to intermittent government supplies. Consequently, several PLHIV and Hypertension spent months without anti-hypertensive medicines. To improve the availability of drugs for non-communicable diseases, the Uganda Ministry of Health had recommended formation of peer support groups. The peer support groups would work by asking members of the support group to collectively raise funds to buy drugs in bulk at a cheaper price. The peer support groups would select an executive committee among the members to support different tasks. The executive committee comprised at least a chairperson, a secretary, and a treasurer. The treasurer was charged with receiving and keeping the members’ financial contributions. Following that consultative performance review meeting in the Integrated HIV/HTN project, healthcare providers mobilized and encouraged several patients to form patient-led peer support groups attached to their health facilities. The health facilities that formed patient-led peer support groups include Ndeija Health center (HC) III, Ntugamo HC IV, Kibaale HC IV, Mugarama HC III, Rwekubo HC IV, Kakoba HC III and Mwizi HC III. We also included Ruhoko HC IV which was not part of the integrated HIV/HTN project but had an active peer support group. A Level IV health facility is overseen by a Medical Doctor and serves a population of 100,000 to 500,000 within its catchment area. These facilities are equipped to handle a wider range of medical needs, including inpatient care with a 30-bed capacity, surgical procedures in an operating theatre, diagnostic testing in a general laboratory, and ongoing management of HIV with a dedicated chronic care unit. In contrast, Level III Health facilities are led by a qualified clinician, nurse, or midwife. They cater to a population of up to 30,000 within their designated area and focus on primary care services. These facilities typically include outpatient departments, maternity wards, and HIV chronic care units. It's important to clarify that peer support groups were not originally included in the integrated HIV/HTN project interventions. However, the project team encouraged health facilities to establish these groups as a complementary strategy to help patients access and adhere to their hypertension medication. Each peer support group typically comprised 5–15 PLHIV who also had hypertension, alongside 10–30 non-HIV individuals with HTN. These groups functioned under a patient-led model, meaning patients managed and facilitated the group discussions and activities. However, the health providers gave oversight and technical support whenever needed. Healthcare providers offered technical support to the peer support groups by recommending appropriate antihypertensive medications and, in some cases, suggesting reliable sources for purchasing these medications. Participation in the peer support groups was entirely voluntary, with patients free to join or leave at any time. For cost estimates, the exchange rate was around 3846 Ugandan shillings (UGX) to 1 US dollar in 2022.

### Data collection procedures

We collected data on basic demographics, and blood pressure (BP) measurements during the survey. Blood Pressure measurement was conducted using sphygmomanometers (Omron M2 Intellisense^™^ Automatic Blood Pressure Monitor, Lake Forest, IL. USA) after an initial period of a seated 5-minute rest. When the first BP measurement reading was normal (< 140/<90 mmHg), no additional BP readings were taken. However, when the first reading was 140/90 mmHg or higher, two additional readings were performed at alternate patient arms three minutes apart, the average BP reading among the three readings would be recorded as final.

### Study Outcomes

The primary outcome was the accessibility of anti-hypertensive drugs through the peer support groups, measured as the number of PLHIV with HTN accessing anti-hypertensives through the peer support groups. Secondary outcomes were participant participation rates in the support groups, measured as the number of PLHIV with HTN willing to join patient clubs and acceptability measured as the number of PLHIV with HTN willing to pool funds in the peer support group to buy drugs.

### Data Analysis

Baseline information was collected and summarized using descriptive statistics. Blood Pressure findings were categorized using the 2020 guidelines of the American Society of Hypertension and the International Society of Hypertension: Normal: <140/90 mmHg; Grade-1 HTN: 140–159/90–99 mmHg; Grade-2 HTN: 160–179/100–109 mmHg; Grade-3 HTN:≥180/110 (25).

## Results

From 3 December 2022 to 20 April 2023, eight out of twenty-six (30.7%) health facilities had established peer support groups for diabetes and hypertension. Health facilities were evenly distributed, with four health facilities at level IV (Ntungamo HC IV, Kibaale HC IV, Ruhoko HC IV and Rwekubo HC IV) and four health facilities at level III (Ndeija HC III, Mugarama HC III, Kakoba HC III and Mwizi HC III).

Out of the 163 PLHIV participants interviewed, 39.3% (64 participants) were members of peer support groups. More than two-thirds (107) of the PLHIV participants received care at Level IV health facilities. There were more PLHIV 58/63 (90.5%) enrolled at Level IV health facilities than at Level III facilities with just 6/63 (9.5%). There was no variation in the number of PLHIV not participating in HTN peer support groups by level of health facility (50.5%) at level III vs 49.5% at Level IV. Slightly over half of the participants 55.2% (90/163) were females, with variation by membership status; 38 (60.3%) were in the HTN peer support group and 52 (52.5%) were not in a peer support group. The mean age for participants was 51.7 years and did not differ by HTN peer support group participation status. Over half of the participants, 97/163 (59.5%) had attained a primary level of education and this did not differ by peer support group participation status; with 38 (23.3%) reporting to have never attended school at all. Most of the PLHIV (99.4%) were on pharmacological HTN treatment, with one participant on the non-pharmacological HTN treatment. Duration of treatment among the participants varied with more than half of the participants having been on treatment for over 6 months.

Overall, 41.1% (67/163) had controlled blood pressure at the time survey and it varied significantly by peer support group participation; with 53.1% (34/64) participants in the peer support group and 33.3% (33/99) participants not in the peer support groups. Nearly all participants (97.5%) had been on ART treatment for more than a year. (Table 1).

### Participation of PLHIV in the peer support groups:

The duration for participation in the HTN peer support varied by months as follows; 6/63(9.5%) for <1 month, 25/63 (40%) for 1-3 months, 21/63 (33%) for 4-6 months, 5/63 (7.9%) for 6-12 months and 6/63 (9.5%) for over 12 months. Among those who participated in peer support groups 57% (36/63) reported accessing anti-hypertensive drugs through the group.

Less than half (44.4%) of participants did not spend on buying HTN medication before joining the peer support group. Whereas one individual (1.5%) spent between UGX10,000 – 30,000 (USD 2.6 – 7.8), 22/63 (34.9%) spent between UGX5,000 – 10,000 (USD 1.3 – 2.6), and 12/63 (19%) spent less than UGX. 5,000 (USD 1.3) averagely per month on buying HTN medications before joining the peer support group.

The amount of money contributed by each PLHIV to the peer support group varied greatly among participants, with 21/63 (33%) contributing UGX. 3,000 (USD 0.8), 29/63 (46%) UGX. 5,000 (USD 1.3), 2/63 (3.2) UGX. 7,000 (USD 1.8), 8/63 (13%) UGX. 10,000 (USD 2.6) and three individuals (1.6%) each contributing UGX. 20,000 (USD 5.2) and above. More than half 38/63 (60.3%) reported the financial contributions made to the peer support group were affordable. Member contributions to the peer support groups were made either once in three months (52%), or monthly (48%). Fifty-five (85.7%) participants provided answers using the Visual Analogue Scale (0-10) regarding satisfaction with their HTN peer support group. The median score was 6, with an interquartile range of 5 - 8. Among those who responded to the satisfaction question, 52/55 (94.5%) reported satisfaction levels of 5 or more. Activities carried out at the HTN peer support group (n=54), included health education, peer counselling, performing blood pressure measurement and group financial savings. (Table 2)

## Discussion

Peer support groups are one of the approaches to involve patients in the self-management of their chronic health conditions (26). This study explored peer support groups for PLHIV with hypertension, where participants provided mutual support in managing their conditions. We evaluated the feasibility and acceptability of patient-led peer support groups in improving access to anti-hypertensive drugs for PLHIV with Hypertension. We found peer support group formation was feasible and PLHIV with Hypertension were able to access antihypertensive drugs through the support groups.

Eight out of twenty-six health facilities formed peer support groups with equal distribution across health facility levels III and IV. Their formation followed a performance review meeting, where it was noted that a significant number of PLHIV with HTN had poorly controlled blood pressure. One of the underlying reasons for not achieving blood pressure control was limited access to hypertension drugs.

Overall, there was low participation (39.3%) of PLHIV in the peer support groups. One of the reasons for low participation in peer support groups was a lack of adequate information about the peer support groups. Others reported challenges in joining because joining the support group required one to make regular financial contributions.

PLHIV with HTN diagnoses who participated in peer support groups accessed anti-hypertensive drugs as compared to those who did not participate. Access to anti-hypertensive medicines is a key component to help patients adhere to treatment. This leads to better management and control of high blood pressure to optimal levels(27). In addition, the PLHIV with HTN who participated in the peer support had better blood pressure control compared to the counterparts who were not involved in the peer support groups. Lowering blood pressure has immense benefits such as preventing strokes, heart failure and coronary heart disease(28).

Furthermore, most respondents in the peer support group noted that the financial contributions to the group were affordable. Most members who participated in the peer support groups contributed $1.3 (UGX 5,000) and below. The amount contributed is comparable to the $1.5 ( 29 ) reported in a similar setting in Kenya. The average expenditure before joining the peer support group was higher, with 36.4% of participants spending an average of $1.3 to $7.8 (UGX 5,000 to UGX30,000). This demonstrates participation in peer support groups could potentially lead to cost savings compared to not participating, although further research is needed to confirm this.

By working together, participants in the peer support groups could explore cost-saving strategies for obtaining antihypertensive medications. These strategies might include negotiating bulk discounts with local pharmacies or identifying relevant government programs. This is supported by a report from the national survey on medicines in Uganda(30) which reported that patient prices for the lowest-priced generics were found to be 2.6 times the international reference price at private retail pharmacies. The collaborative approach within the peer support groups resembles a micro-insurance model where all the participants are assured of hypertension medicines as long as they make a financial contribution. And that contribution was not dependent on the type of medicines a patient is on or the severity of the disease. In the care of hypertension, some patients can be initially on a lifestyle modification plan, or pharmacological depending on the level of blood pressure reading. Those on the pharmacological plan may be on 1–3 types of medication(9). So irrespective of the number of hypertension drugs one is taking they all still contribute the same amount. The patients not on treatment also benefit by receiving health education on lifestyle changes offered in the peer support groups.

Most participants reported high levels of satisfaction with the performance of the peer support groups. This demonstrated that peer support groups for PLHIV with hypertension can benefit participants at the individual level. Activities carried out at the peer support group were health education, peer-to-peer counselling, and performing blood pressure measurements. These activities form part of and enhance the self-management approaches(31). Education on hypertension is a key strategy for patients and has been demonstrated to improve patients’ understanding of their disease state and treatment(32).

## Conclusion

We found that patient-led peer support groups were a feasible intervention to improve access to essential medicines for PLHIV with HTN in rural settings in Uganda. Our results demonstrate that peer support groups can be leveraged to support patient groups struggling with limited access to a supply of medicines. Further studies are recommended to evaluate the effectiveness of peer support groups on HTN control among PLHIV.

## Figures and Tables

**Table 1:** Sociodemographic and clinical characteristics in N (%), unless noted, of Persons Living with HIV with hypertension who participated in the Cross-sectional survey conducted in Southwestern Uganda from December 2022 to April 2023.

Characteristics	Peer Support Group	Not in Peer Support Group	Total
	N=63	N=99	N=163
Health Facility			
Level III	6 (9.5%)	50 (50.5%)	56 (34.4%)
Level IV	58 (90.5%)	49 (49.5%)	107 (65.6%)
Female, Sex	38 (60.3%)	52 (52.5%)	90 (55.2%)
Age, Mean [SD]	52.6 (10.1)	51.1 (12.4)	51.7 (11.6)
Education			
No school	11	27	38(23.3%)
Primary	42	55	97 (59.5%)
Secondary	6	8	14 (8.6%)
Tertiary/Vocational	5	5	10 (6.1%)
University	0	3	3 (1.8%)
Post-graduate	0	1	1 (0.6%)
ART Treatment duration			
Less than a month			
1-3 months	0	1	
4-6 months	0	1	
6-12 months	1	1	
5: More than a year ago	63	96	159 (97.5%
Hypertension Treatment			
Pharmacological	64	98	162 (99.4%)
Non-pharmacological	0	1	1 (0.6%)
Hypertension treatment duration			
Less than a month			
1-3 months	1	2	3 (1.8%)
4-6 months	9	16	25 (15.3%)
6-12 months	10	15	25 (15.3%)
5: More than a year ago	9	24	33 (20.2%)
	35	42	77 (47.2%)
Blood pressure control	34 (53.1%)	33 (33.3%)	67 (41.1%)

**Table 2:** Number and proportion of Persons Living with HIV who attended peer support groups and participated in the Cross-sectional survey conducted in Southwestern Uganda from December 2022 to April 2023.

Duration of participation in the peer support group	N=63		%
<1 month	6		9.5
1-3 month	25		40
4-6 month	21		33
6-12 month	5		7.9
>12 months	6		9.5

The average amount spent monthly buying HTN medications before joining peer support group	N=63		%
< $1.3 (<UGX5,000)	12		19.0
$ 1.3 – 2.6 (UGX 5,000-10,000)	22		34.9
$2.6 −7.8 (UGX10,000-30,000)	1		1.5
Not spent	28		44.4

The actual amount contributed to the peer support group (Uganda shillings)	N=63		%
$0.8 (UGX 3,000)	21		33
$1.3 (UGX 5,000)	29		46
$1.8 (UGX 7,000)	2		3.2
$2.6 (UGX10,000)	8		13
$5.2 (UGX 20,000)	1		1.6
$6.5 (UGX 25,000)	1		1.6
$7.8 (30,000)	1		1.6

Accessibility to HTN drugs through the peer support group	N=63		%
Yes	36		57
No	27		43

Affordability of the member contributions	N=63		%
Yes	38		60.3
No	25		39.7

Frequency of contributions	n=48		%
once a month	23		48
once in 3 months	25		52

Patient satisfaction with the performance of the Peer support group	VAS Scale	VAS Scale scores	%
	1	0	0
	2	1	1.8
	3	1	1.8
	4	1	1.8
	5	20	36
	6	6	11
	7	7	13
	8	7	13
	9	8	15
	10	4	7.3

Activities carried out in the Peer support group	N=61		%
Health education	34		56
Health education & performing BP	4		6.6
Health education, performing BP & peer counselling	3		4.9
Health education & peer counselling	18		30
Health education & group financial savings	2		3.3

## Data Availability

The authors confirm that the data supporting the findings of this study are available with the article. Raw data that supports the findings of this study are available from the corresponding author upon request.
